# Higher-level cognitive functions in Dutch elite and sub-elite table tennis players

**DOI:** 10.1371/journal.pone.0206151

**Published:** 2018-11-07

**Authors:** Marije T. Elferink-Gemser, Irene R. Faber, Chris Visscher, Tsung-Min Hung, Sjoerd J. de Vries, Maria W. G. Nijhuis-Van der Sanden

**Affiliations:** 1 Center for Human Movement Sciences, University Medical Center Groningen, University of Groningen, Groningen, The Netherlands; 2 Institute of Sport Science, University of Oldenburg, Oldenburg, Germany; 3 International Table Tennis Federation, Lausanne, Switzerland; 4 Department of Physical Education, National Taiwan Normal University, Taipei City, Taiwan; 5 Faculty of Human Resource Management and Applied Psychology, Saxion University of Applied Sciences, Deventer, The Netherlands; 6 Radboud university medical centre, Radboud Institute for Health Sciences, IQhealthcare, Nijmegen, The Netherlands; Universita degli Studi di Verona, ITALY

## Abstract

This study aimed to investigate the higher-level cognitive functions (i.e. metacognition and executive functions) of Dutch competitive table tennis players to better understand its relevance for performance in this fast and complex sport. Thirty elite (age 16 ± 4) and thirty age and sex-matched sub-elite peers (age 16 ± 5) were assessed on metacognition and executive functions (working memory, inhibitory control, cognitive flexibility) using D-KEFS tests. Compared to norm scores, both the Dutch competitive elite and sub-elite table tennis players scored above average on all tests (*p* < 0.05). MANOVA showed a main effect for performance level (elites outscored sub-elites; *p* < 0.05). T-tests revealed that elite players make less mistakes on tests for inhibitory control (CWI-3: 0.9 ± 0.9; CWI-4: 1.1 ± 1.2) than sub-elite players (CWI-3: 1.8 ± 1.1; CWI-4: 2.6 ± 1.5) (*p* < 0.05). When controlling for training hours in a MANCOVA, no significant main effect of performance level remained (*p* > 0.05). In conclusion, Dutch elite and sub-elite table tennis players are characterized by above-average scores on higher-level cognitive functions compared to norm scores. A relation with performance level has been shown, which may be explained by the greater exposure to table tennis for elite compared to sub-elite players. However, longitudinal research is needed to indicate the direction of this association.

## Introduction

Higher-level cognitive functions, such as metacognition and executive functions, are involved in the control and regulation of “lower-level” cognitive processes, such as reaction time, enabling goal-directed, future-oriented behaviour [1,2]. Metacognition reflects “the use of strategies that are thoughtfully brought to mind as one prepares to solve a problem and then a monitoring of progress towards a specific goal” [3]. It includes the three core executive functions, working memory, inhibitory control and cognitive flexibility. Working memory refers to the ability of holding information in mind and manipulating this information. The ability to stay concentrated, ignore distractions, and resist a response in favour of another, better response refers to ‘inhibitory control’ requiring ‘attention’. Finally, the ability to adapt quickly to new demands and rules based on fast changing situations reflects someone’s ‘cognitive flexibility’ [2,4,5].

Currently data on the relationship between sport expertise and general cognitive abilities is mixed [6], and there is no hard evidence that expert athletes have superior basic cognitive abilities compared to normal, physically active controls or even that they are a limiting factor of sport performance [7,8]. Even so, the importance of higher-level cognitive functions for performance in open-team sports, such as soccer, has been shown in several studies [9,10]. Better performing players outscored lesser performing players on a variety of higher-level cognitive function tests and a relation has been suggested with sport-specific skills such as better positioning and decision-making in the field and scoring a goal [11]. Open-team sports involve interaction with direct opponents as well as with teammates and as such contain situations with high amounts of unstable and seemingly unpredictable events [12]. Although table tennis is different from an open-team sport, since it is primarily an individual sport (with the exception of doubles) players are still faced with continuously changing conditions. In order to excel, players dedicate much of their time and effort to table tennis spending many hours in training. However, still a lot is unknown about what the best training practices are. Insight in which factors relate to performance gives valuable directions for the content of training sessions. Consequently, an investigation into the role of metacognition and executive functions for table tennis performance could prove insightful [13]. One way to do so, is to compare players of different levels of performance with each other.

The interaction with a direct opponent is an essential ingredient of table tennis. Sports with direct opponents are said to challenge the brain in that they contain demanding activities which require a higher-level cognitive function in order to be successful [14]. Even more, table tennis is one of the fastest, if not the fastest, ball-sports in the world leaving little time for a player to return the ball [15,16]. Players alternately and repeatedly hit a 2.7g ball on a relatively small table controlling the spin, velocity and placement of the ball in order to make it difficult for opponents to successfully return it [17]. This means that to perform well, a player needs to handle severe time constraints while anticipating the opponent’s intentions, recognizing meaningful cues in the context of the game, deciding in a split second which action to make, and initiating an appropriate response [18,19]. Making mistakes while returning the ball results in points won by the opponent during the rally, and consequently it becomes more difficult to win the game. Considering these task constraints within the game of table tennis, it seems reasonable that higher-level cognitive are also important for performance in table tennis. Indeed, a recent study on attention showed differences between adolescent table tennis players and non-players in favour of the table tennis players [20]. Similarly, Hung and colleagues found that compared to non-athlete controls, elite table tennis players were able to maintain superior reactivity when facing situations with different degree of uncertainty [21]. Moreover, regular adolescent tennis players outperformed individual closed sport athletes (i.e. swimmers) and non-athletes on inhibitory control [22] although this was investigated in regular tennis and not in table tennis. To the best of our knowledge, so far there are no studies available about higher-level cognitive functions in table tennis players.

To investigate whether competitive table tennis players are indeed characterized by above-average scores on higher-level cognitive functions, the first goal of this study is to compare elite and sub-elite table tennis players on higher-level cognitive functions with age-related norm scores. Our hypothesis is that the competitive table tennis players perform above average. To better understand the relevance of higher-level cognitive for performance in table tennis, the second part of this study will focus on comparing elite and sub-elite table tennis players while controlling for potential effects of sex and age. Our hypothesis is that elite table tennis players outperform sub-elite table tennis players on all tests for metacognition and the executive functions. To further unravel the relation with performance level, it will be investigated whether potential differences between elite and sub-elite players remain when controlled for accumulated training hours.

## Methods

### Participants

In total, 60 Dutch competitive table tennis players participated, from which 30 were considered elite and 30 sub-elite. Elite players were recruited from the national selections of the Netherlands Table Tennis Association. All eligible elite players had the highest national tournament license (A-license). Metacognition and executive functions may be related to sex and age. To control for this, each of the 30 elite players was matched with a sub-elite player on the basis of sex and age by using the national ranking. In this way, 30 pairs of one elite and one sub-elite player were formed. Sub-elite players needed to train and play competition at a table tennis club joining the Netherlands Table Tennis Association and have a national tournament license that was at least one level lower than the elite players (B-, C-, or D-license). The study protocol and informed consent procedure were approved by the Ethics Committee of the Medical Spectrum Twente (Medical School Twente, Institute for Applied Science, Enschede, the Netherlands; METC/13053.fab 19-2-2013) and performed in full compliance with the Declaration of Helsinki. Written informed parental consent and player assent was obtained for all participants under the age of 18 years. Written informed consent was obtained for all adult participants.

### Metacognition and executive functions

To measure metacognition and executive functions, various measures of the Delis-Kaplan Executive Function System (D-KEFS, 2001) were obtained: The D-KEFS Design Fluency Test (DFT), D- KEFS Colour-Word Interference test (CWI), and the D-KEFS Trail Making Test (TMT) [23]. Validity and reliability are reported to be satisfactory for all executive measures with a sound technical adequacy for a wide application in educational and clinical settings [24,25]. Evidence for validity has been reported by 1.) correlations of measures within and between individual D-KEFS test, 2.) correlations of the D-KEFS tests with other cognitive tests including both evidence for convergent and discriminant validity of the tests and 3.) findings from studies with clinical populations presenting sensitivity to distinguish differences in performance levels. The internal consistency (CWI 0.62–0.86; TMT 0.68–0.79), test-retest correlations (DFT 0.13–0.58; CWI 0.49–0.90; TMT 0.36–0.82) and standard errors (DFT 1.94–2.47; CWI 1.28–1.85; TMT 1.38–1.69) were considered adequate (i.e. moderate) to good and comparable to other commonly used neuropsychological tests (e.g. the Wisconsin Card Sorting Test and the Wechsler Memory Scale-III) [23–25]. All players were assessed at their training centre. The tester was a psychology student who was well-trained in using the test protocols of the manual. Instruction and feedback was given during extensive training by a professional neuroscientist.

#### D-KEFS Design Fluency Test (DFT)

This test measures metacognition; a combination of a player’s working memory, inhibitory control, and cognitive flexibility. It comprises three conditions: filled dots, empty dots, and switching. In each condition, a player is instructed to make as many different designs as possible in each square by connecting dots using only four straight lines in 60 seconds. Outcome measure for all conditions is the number of correct designs with a higher score being a better score. No credit is given if the design contained greater or fewer than four lines or was a repetition of a previous design. In the first condition (DF-1), the player is presented squares containing five filled (i.e., black) dots, and is asked to draw their designs by connecting the dots. In the second condition (DF-2), the squares contained five empty dots and five filled dots. A player is instructed to connect only the empty dots. In the third and final condition (DF-3), the squares also contained five empty dots and five filled dots; however, in this condition, the player is instructed to alternate between connecting empty dots and filled dots. No credit is given for designs in which participants does not switch correctly. For the D-KEFS DFT, the primary outcome measure for metacognition is the total number of correct patterns from all three conditions (DFT total = DFT-1 + DFT-2 + DFT-3). For each condition, raw scores as well as scaled scores are noted.

#### D-KEFS Colour-Word Interference test (CWI)

This test measures a player’s ability to inhibit automatic responses and cognitive flexibility. There are four conditions. In all conditions, the player is instructed to work as fast as possible without making mistakes. Outcome measures are (1) the time in seconds whereby a lower score denotes a better score (i.e., less time to complete the test) and (2) the number of mistakes made, whereby a lower score means a better score (i.e., less mistakes made). The first two conditions measure a player’s colour naming ability and basic reading ability. In CWI-1, a player names the colour of printed ink patches. In CWI-2, a player reads the colour of a word printed in black ink. In CWI-3, a player is instructed to name the colour of the ink of a printed word while inhibiting reading the conflicting word. In this condition, inhibitory control is measured. In CWI-4, a player must alternate between reading the word and saying the ink colour. In this condition, a combination of inhibitory control and cognitive flexibility is measured. To control for colour naming ability in D-KEFS CWI, two contrast scores are calculated (CWI-contrast 1: inhibition vs colour naming ability CWI-3 minus CWI-1, and CWI-contrast 2: inhibition/cognitive flexibility versus colour naming ability CWI-4 minus CWI-1). In each condition, raw scores as well as scaled scores are noted.

#### D-KEFS Trail Making Test (TMT)

This test measures a player’s cognitive flexibility. There are five conditions. In all conditions, the player is instructed to work as fast as possible without making mistakes. Outcome measures for all conditions are the time in seconds whereby a lower score denotes a better score (i.e. less time to complete the test). For the first four conditions the number of mistakes made is an outcome measure, in which a lower score means a better score (i.e. less mistakes made). The first three conditions measure a player’s visual scanning -, number sequencing -, and letter sequencing abilities. In TMT-1, a player needs to find all numbers ‘3’ on a page of numbers and letters. In TMT-2, a player must connect numbers with each other starting with ‘1’, connecting to ‘2’, then ‘3’ and so on, on a page of numbers and letters. In TMT-3, a player is asked to connect only the letters with one another starting with ‘A’, connecting to ‘B’, then ‘C’, and so on, on a similar page of numbers and letters. In TMT-4, a player must alternate between numbers and letters by connecting first ‘1’ to ‘A’, connecting to 2, then ‘B’, and so on. In this condition, cognitive flexibility is measured. In TMT-5, motor speed is measured. A player is asked to draw as fast as possible a line on a variety of dotted lines. To control for visual scanning-, number sequencing -, and letter sequencing ability as well as for motor speed in D-KEFS TMT, four contrast scores are calculated (TMT-contrast 1: cognitive flexibility versus visual scanning TMT-4 minus TMT-1, TMT-contrast 2: cognitive flexibility versus number sequencing TMT-4 minus TMT-2, TMT-contrast 3: cognitive flexibility versus letter sequencing TMT-4 minus TMT-3, and TMT-contrast 4: cognitive flexibility versus motor speed TMT-4 minus TMT-5). For each condition, raw scores as well as scaled scores are noted.

### Table tennis performance

Competition rating scores from The Netherlands Table Tennis Association’s archives were obtained for each player. These scores indicate the player’s individual competition performance at the moment of testing. A higher rating score denotes a better player’s table tennis performance. The competition rating score allows to compare performances between players (youth and adult players, male and female players) who participate in any of the regional and national competition leagues [26]. Besides the competition rating score, playing hand (right or left handedness), the current number of training hours per week and the accumulated training amount (hours) were acquired by using a short questionnaire. Playing hand was determined by the hand a player used as playing hand during table tennis. To determine the accumulated training hours, the players were first asked to fill in the average number of training hours per week in for all years they played table tennis. The outcome was multiplied by the average number of training weeks that year. Thereafter, the average number of training hours per week was asked for one year prior as well as the average number of training hours that year. Both questions were repeated until the year of entry in competitive table tennis. The accumulated training amount in hours was calculated by summing up the yearly training volume of all years. Table 1 shows personal as well as table tennis related characteristics of the players.

**Table 1:** Personal and table tennis related characteristics of Dutch elite and sub-elite table tennis players (n = 60).

	Total (n = 60)	Elite players (n = 30)	Sub-elite players (n = 30)
Sex (male:female)	24:36	12:18	12:18
Playing hand (right:left)	47:13	26:4	21:9
Age (years)	15.8 (4.3)	15.6 (3.6)	15.9 (5.0)
Training (hours/week)	8.9 (7.1)	14.5 (6.2)*	3.8 (2.4)*
Accumulated training amount (hours)	2442 (2399)	3924 (2803)*	1124 (526)*
Performance rating (points)	1353 (572)	1734 (422)*	971 (433)*

Sex and playing hand are frequencies. Age, training and accumulated training amount are presented in means (SD).

*Significant difference between elite and sub-elite table tennis players (P < 0.001).

### Statistical analysis

Data were analysed using IBM SPSS Statistics 20.0.0 (IBM Corp. Somers, NY). Raw scores of each player were converted to scaled scores by comparing player’s scores to age-related norm scores which were based on a representative, stratified sample of 1750 children, adolescents, and adults aged 8–89 from the United States of America. One-sample T-tests were conducted to compare the scaled scores of the total group as well as for elite and sub-elite table players separately with norm scores (M = 10, SD = 3) on each of the test conditions. A MANOVA was performed with raw scores for metacognition and executive functions (DFT-total, CWI-3 time, CWI-3 mistakes, CWI-4 time, CWI-4 mistakes, TMT-4 time, TMT-4 mistakes) as dependent variables, and performance level (elite versus sub-elite) as between-subjects’ independent variable. T-tests were executed as follow-up tests to analyse differences between elite and sub-elite table tennis players for each test condition separately. To further unravel any differences between both performance groups, a MANCOVA was executed with raw scores for metacognition and executive functions as dependent variables, performance level as independent variable and accumulated training amount in hours as covariate. Statistical significance with two-sided testing was accepted at *p* < 0.05. To interpret the results, Cohen’s d effect sizes (d) were calculated between the elite and sub-elite table tennis players. An effect size around 0.20 is considered small, around 0.50 medium and around 0.80 large [27].

## Results

### Dutch competitive table tennis players versus norm population

In Table 2, scaled scores on the DFT, CWI, TMT of elite and sub-elite table tennis players are compared to norm scores and t-values as well as p-values are provided. Results from the DFT for metacognition (working memory, inhibitory control, cognitive flexibility) show that competitive table tennis players score above average on all conditions (*p* < 0.05). This is the case for elite as well as sub-elite players. Competitive table tennis players, either elite or sub-elite, perform above average on the CWI test for inhibition. They need less time to complete the test and make less mistakes than their peers according to the norm scores (*p* < 0.05). These results remain when the players’ colour naming ability is controlled for. Competitive table tennis players also score above average on the CWI test for a combination of inhibition and cognitive flexibility. Only for elite players, this result remains when controlling for colour naming ability (*p* < 0.05). In addition, unlike sub-elite players, compared to the open population, elite players make less mistakes than average (*p* < 0.05). Results from the TMT show a more complex pattern. Competitive table tennis players score above average on cognitive flexibility, they need less time to complete the test and make less mistakes (*p* < 0.05). Based on post analysis, this was connected to visual scanning ability and motor speed. The players score below average when controlling for visual scanning ability and motor speed (*p* < 0.05). When controlling for number sequencing -, and letter sequencing ability competitive table tennis players score similar to the average on cognitive flexibility (*p* > 0.05). This holds for elite as well as sub-elite players.

**Table 2:** Scaled scores on D-KEFS tests for metacognition and executive functions (mean (SD)) of Dutch elite and sub-elite table tennis players (n = 60) compared to norm scores (M = 10, SD = 3).

	Total (n = 60)	t-value / *p*-value	Elite players (n = 30)	t-value / *p*-value	Sub-elite players (n = 30)	t-value/ *p*-value
*Design Fluency Test for metacognition (working memory*, *inhibitory control*, *cognitive flexibility)*						
DFT-1	13.1 (2.6)*	9.054/ < .0001	13.4 (2.6)*	7.208/ < .0001	12.3 (2.7)*	5.619/ < .0001
DFT-2	13.9 (2.4)*	12.702/ < .0001	14.4 (2.5)*	9.641/ < .0001	13.4 (2.2)*	8.517/ < .0001
DFT-3	13.0 (2.7)*	8.536/ < .0001	13.1 (2.7)*	6.127/ < .0001	12.8 (2.6)*	5.857/ < .0001
DFT-total	13.9 (2.4)*	12.554/ < .0001	14.5 (2.5)*	9.963/ < .0001	13.3 (2.2)*	8.118/ < .0001
*Colour Word Interference Test for inhibitory control and cognitive flexibility*						
CWI-3 Time	11.6 (2.0)*	6.299/ < .0001	11.7 (2.1)*	4.594/ < .0001	11.5 (1.9)*	4.253/ < .0001
CWI-contrast 1	11.1 (1.9)*	4.561/ < .0001	11.4 (2.0)*	3.971/ < .0001	10.8 (1.8)*	2.449/.021
CWI-3 Number of mistakes	11.6 (1.5)*	7.972/ < .0001	12.1 (1.3)*	9.332/ < .0001	11.0 (1.6)*	3.476/.002
CWI-4 Time	11.6 (2.2)*	5.668/ < .0001	12.2 (1.9)*	6.145/ < .0001	11.1 (2.4)*	2.498/.018
CWI-contrast 2	11.2 (2.5)*	3.637/.001	11.9 (1.9)*	5.542/ < .0001	10.4 (2.8)	.790/.436
CWI-4 Number of mistakes	11.0 (1.6)*	4.923/ < .0001	11.8 (1.3)*	7.737/ < .0001	10.3 (1.6)	.928/.361
*Trail Making Test for cognitive flexibility*						
TMT-4 Time	11.2 (2.4)*	3.724/ < .0001	11.1 (2.2)*	2.697/.012	11.2 (2.6)*	2.549/.016
TMT-contrast 1	8.6 (1.7)*	-6.527/ < .0001	8.5 (1.8)*	-4.629/ < .0001	8.7 (1.6)*	-4.551/ < .0001
TMT-contrast 2	10.2 (1.9)	.961/.341	10.1 (1.9)	.283/.779	10.4 (1.8)	1.087/.286
TMT-contrast 3	9.8 (1.9)	-.947/.347	9.6 (1.8)	-1.087/.286	9.9 (2.0)	-.275/.785
TMT-contrast 4	8.4 (2.3)*	-5.357/ < .0001	8.2 (2.3)*	-4.208/ < .0001	8.6 (2.3)*	-3.325/.002
TMT-4 Number of mistakes	11.0 (1.4)*	5.252/ < .0001	10.7 (1.7)*	2.391/.024	11.2 (1.1)*	6.000/ < .0001

*Significant difference with norm score (p < 0.05).

### Dutch elite versus sub-elite table tennis players

In Table 3, raw scores on the DFT, CWI, and TMT are presented for elite and sub-elite table tennis players. The results of the MANOVA showed a main effect for performance level (Wilks’ lambda = 0.662, F(10,49) = 2.505, *p* = 0.016). T-tests revealed significant differences between elite and sub-elite players on the number of mistakes on the CWI-3 (F (1,57) = 5.651, *p* = 0.021) and CWI-4 (F (1,57) = 8,275, *p* = 0.006), with elite players making fewer mistakes than sub-elite players. On the CWI, effect sizes ranged from small to medium on the time scores and were large on the number of mistakes. On most variables, small-to-medium effect sizes were found with the trend that elite players have better scores than sub-elite players. No significant differences were found between the two groups for any of the other variables, either on the DFT, CWI, or TMT (*p* > 0.05). The results of the MANCOVA, with accumulated training amount in hours as covariate, showed no main effect for performance level (Wilks’ lambda = 0.757, F(10,48) = 1.540, *p* = 0.155).

**Table 3:** Raw scores on D-KEFS tests for metacognition and executive functions (mean (SD)) of Dutch elite and sub-elite table tennis players (n = 60).

	Elite players (n = 30)	Sub-elite players (n = 30)	F-value / *p*-value	Effect size d
*Design Fluency Test for metacognition (working memory*, *inhibitory control*, *cognitive flexibility)*				
DFT-1	13.3 (3.2)	12.4 (3.0)	1.355 / .249	0.29
DFT-2	15.4 (3.3)	14.1 (3.0)	2.585 / .113	0.41
DFT-3	10.2 (2.6)	9.9 (2.3)	.268 / .606	0.12
DFT-total	40.9 (6.1)	39.0 (6.0)	1.459 / .232	0.31
*Colour Word Interference Test for inhibitory control and cognitive flexibility*				
CWI-3 Time (s)	47.9 (9.3)	49.6 (11.1)	.397 / .531	0.17
CWI-3 Number of mistakes	0.9 (0.9)*	1.8 (1.1)*	12.257 / .001	0.90
CWI-4 Time (s)	52.4 (9.2)	57.5 (12.0)	3.405 / .070	0.48
CWI-4 Number of mistakes	1.1 (1.2)*	2.6 (1.5)*	18.392 / < .001	1.10
*Trail Making Test for cognitive flexibility*				
TMT-4 Time (s)	60.2 (17.3)	61.7 (36.1)	.042 / .838	0.05
TMT-4 Number of mistakes	0.7 (1.0)	0.4 (0.6)	1.485 / .228	0.36

*Significant difference between elite and sub-elite table tennis players (p < 0.05)

d around 0.20 is small; d around 0.50 is medium; d around 0.80 is large (Cohen, 1988).

## Discussion

To investigate whether Dutch competitive table tennis players are characterized by above-average scores on higher-level cognitive functions and to understand its relevance for performance, elite and sub-elite players’ metacognition and executive function scores were compared to norm values of their peers of the open population. In addition, a comparison was made between elite players and age and sex-matched sub-elite peers on metacognition, inhibitory control, and cognitive flexibility. Compared to norm scores, the competitive table tennis players scored above average on all tests. The results revealed that elite players make less mistakes on tests for inhibitory control than sub-elite players. When controlling for accumulated training hours, the significant difference between elite and sub-elite players on the combination of the various higher-level cognitive functions disappeared. These results are largely in line with the hypotheses.

Examples of situations in which higher-level cognitive functions are essential are those that require concentration, problem solving, change, choices among alternatives and overriding a strong internal or external pull [28]. Competitive table tennis players are in these kinds of situations on a regular basis in their training and when playing games. As such, it is not remarkable that they have higher scores than the open population on tests for metacognition and executive functions. Given the severe time-constraints of the game, players need to plan, anticipate, decide and execute their actions fast, appealing to well-developed high-level cognitive functions [14]. Similar results have been reported with national-level soccer players [11]. These players scored relatively better than the competitive table tennis players from the current study, with their results placing them in the top 5% of the population. A possible explanation may be that soccer, even more so than table tennis, is a sport which requires high-level cognitive functions due to the influence of teammates as well as opponents.

Noticeable are the results of the current study on the Trail Making Test. When analysing the different test conditions, it appears that the table tennis players score higher on cognitive flexibility than the norm population. However, they score lower than the norm population when their cognitive flexibility score is controlled for both visual scanning and motor speed. This is not the case when controlled for either their number sequencing- or letter sequencing ability. As such, table tennis players seem to score high on this test for cognitive flexibility, not because of their well-developed cognitive flexibility, but merely because of their high scores on visual scanning as well as motor speed, which are lower-level cognitive functions. This may be explained from the task constraints of table tennis. During a game, players need to attend the most meaningful yet minimal cues under severe time-constraints. Visual scanning of the small ball’s position to the table, its possible flight and the position and intention of the opponent seems essential, in combination with producing fast effective strokes. This is supported by research in badminton, another racket sport in which players have to respond to visual stimuli under critical time pressure. From a comparison between skilled players and age-matched nonathletic controls it was concluded that visual motion processing sub serves faster visuomotor reaction in badminton players [29]. The neurophysiological process contributing to expert visuomotor performance in badminton players has been investigated as well, underlining the importance of fast visuomotor transformation in the premotor and supplementary motor cortical regions [30]. During training sessions, players may rehearse pre-determined patterns extensively. It is likely that the Dutch elite/sub-elite players from this study, most of whom are not international top-level players, are characterized by playing in more-or-less ‘fixed’ patterns on how to return the ball, requiring well-developed visual scanning and motor speed, but less cognitive flexibility. Although cognitive flexibility seems particularly important in situations which are less predictable, for example in open team sports with a high degree of freedom in choices for teammates and opponents [31,32], this does not mean it may not be characteristic for expert table tennis players from the highest international level of performance. So far, literature is lacking on this topic, but if future studies confirm that this is the case, valuable input for table tennis players could be provided to not only rehearse fixed patterns during training and games. Instead, players should also be challenged to be flexible in how to return the ball. However, more research is warranted before underpinned practical suggestions for trainers can be made.

The multivariate analysis showed a main effect for level of performance, elite players had higher scores than the sub-elite players on the combined construct of higher-level cognitive functions. With trends on most variables in the expected direction, elite players scored particularly better on inhibitory control than sub-elite players. The better performing players clearly distinguished themselves from their sub-elite counterparts by the smaller amount of mistakes they made while executing the CWI test. For table tennis, it seems crucial to be able to minimize the number of mistakes while playing at high speed, since every mistake directly results in a point won by the opponent, and consequently the chances of winning games. This is reflected in that the elite players in the current study were more successful than the sub-elite players; the elites had higher ratings than the sub-elites (Table 1). Similar results have been reported in talented soccer players [33]. To be in control during a game, not only speed is of utmost importance. The precision of executing technical skills (i.e., making less mistakes) is essential as well. Many studies demonstrated that there is a relation between the speed of execution of skills and the number of mistakes made [34]. This phenomenon is explained in motor control studies. The so-called speed-accuracy trade-off states that the quicker motor skills are performed, the greater the increase in errors is [35]. In the study on talented soccer players, sub-elite players were equally fast on a field-test for passing the ball, but made significantly more mistakes than elite players. They had more difficulty in remaining accurate at high speeds [33].

The current study included table tennis players from a wide age-range. With the exception of the studies by Huijgen et al. (2015) and Verburgh et al. (2014), most studies on the relation between higher-level cognitive functions and sport performance focused on adolescent or adult players [9,10]. However, in terms of talent identification and talent development, it seems essential to acquire knowledge on youth players as well, preferably applying a multidimensional, longitudinal design [36,37]. Elementary forms of executive functions are present as early as the preschool period (3–5 years) and develop from early childhood through adolescence into young adulthood [28,38,39].

Players may be more successful in table tennis partly because of their better higher-level cognitive functions. However, the opposite may also be true, i.e., higher cognitive functions are the result of more playing experience. As a task is learnt, decision-making improves with practice [40]. When becoming more successful, players spend more time to table tennis, leading to a greater accumulated amount of training. In several studies, the development of executive functions has been related to physical exercise [41,42]. Being exposed to high volumes of table tennis training may improve players’ higher-level cognitive functions. From Table 1 it becomes clear that there are differences between elite and sub-elite players in terms of table tennis training. This may have influenced the results in favour of the elite table tennis players, which is supported by the results from the MANCOVA. When controlling for accumulated training hours, differences between elite and sub-elite table tennis players disappeared suggesting that exposure to table tennis explains the better scores on the combined construct of higher-level cognitive functions of elite players. The number of training hours is likely to be positively related to players’ physical fitness. While most of the literature has demonstrated the beneficial influence of aerobic fitness on execution functions in older adults [43,44], recent studies have also linked executive control functions to physical activity among children [45,46]. As such, differences on cognitive tasks may have been caused by differences in fitness levels. In this regard, Chaddock and colleagues (2011) recommend to include a high-fit age-matched group in studies comparing cognitive functions of different groups [47].

Several suggestions for future research could be derived from the present study that need to be acknowledged. First, the choice for the cognitive function tests in the current study is not without limitations. In a review, Cremen and Carson (2017) explore whether standard tests of cognitive function are misappropriated in the study of cognitive enhancement [48]. This may also apply on sport studies. Originally, the D-KEFS has been designed for clinical disorders. Although validity and reliability have been reported to be satisfactory for all executive measures [24,25], the D-KEFS has also been criticized based on its psychometric properties [49]. In addition, it is highly relevant to consider critically the nature of outcome measures used in relation to table tennis performance. Ecological validity is frequently defined as the extent to which results obtained in controlled experimental settings apply to real-world naturalistic settings [50]. The point is that it remains important to keep this in mind when interpreting the results of the current study. Second, considering the relative small sample size of the current study, there is a risk of false-positive results. Although it is a true challenge to include high numbers of elite and sub-elite athletes, it is recommended to increase sample size, for example by combining players from multiple countries, and consequently the power of the analyses. Third, future studies are also advised to adopt a longitudinal design to provide more convincing evidence for a causal relationship between higher-level cognition and table tennis performance, which is not possible from the present study due to its cross-sectional design. Fourth, future research should control for possible confounding variables such as IQ, educational status / academic achievement, socio-economic status, and physical fitness. A positive association of childhood cardiorespiratory fitness (CRF) with cognitive function has been reported by several studies [51]. As such it is recommended that future research includes CRF as an explicit covariate to reduce its confounding effect. Moreover, in addition to the positive relation between the higher-level cognitive functions measured in this study and table tennis skill levels, future research may want to look at other cognitive functions as well. Specifically, visuo-spatial cognition could be one of the target variables for this endeavor. Table tennis players, like athletes from the majority of sports, are required to place high demands on visuo-spatial processing during their performance [52]. Evidence from studies in table tennis and other, related open-skills sports have shown the relevance of visuo-spatial cognition. For example, table tennis players exhibited better performance relative to non-athletes when they performed the attentional network test [20]. Similarly, badminton athletes exhibited shorter reaction times in both visuo-spatial attention and memory conditions than non-athletes [53]. A recent study also found that both closed- and open-skilled athletes outperformed non athletes in a task with a high demand on visuo-spatial processing [54].

In conclusion elite and sub-elite table tennis players are characterized by above-average scores on several higher-level cognitive functions. A relation with performance level has been shown, which may be explained by the difference in exposure to table tennis between elite and sub-elite players. However, longitudinal research is needed to indicate the direction of this association.
